# DIFFERENCES OF RANKINGS IN DEATH CERTIFICATES AMONG PERSONS WITH ALZHEIMER’S DISEASE

**DOI:** 10.1093/geroni/igae098.3614

**Published:** 2024-12-31

**Authors:** Candace Brown, Yinghao Pan, Maggi Miller, Matthew Lohman

**Affiliations:** University of North Carolina Charlotte, Charlotte, North Carolina, United States; University of North Carolina Charlotte, Charlotte, North Carolina, United States; University of South Carolina, Columbia, South Carolina, United States; University of South Carolina, Columbia, South Carolina, United States

## Abstract

A 2021 report indicates that 19.1% of people ages 76-84 and 34.6% of people aged 85 and older have Alzheimer’s disease (AD). Data provided on death certificates using the International Statistical Classification of Diseases is guided by the WHO for coding cause and contributing comorbidities of death. Death rankings assist in monitoring which diseases need attention for better informed decision making in healthcare. However, classifying immediate cause of death presents challenges when the likelihood of polymorbidity occurs with advancing age in patients with presenting AD but inconclusive diagnoses of the disease. We used the Alzheimer’s Disease and Related Dementia South Carolina (SCADR) registry and linked it with state death certificates to examine sociodemographic variables and survival probabilities of decedents between the years 2014-2019. Cox proportional hazards models were fitted and hazard ratios (HRs) computed, adjusting age at diagnosis and race. Turnbull’s method estimated the survival probabilities or curves for subgroups as the time from AD diagnosis to death. Of the 78,534 patients in the study 94% were diagnosed with AD at age 61 or older. AD was listed as the immediate cause of death among all racial groups, except for Native American/American Indian, contrasting from previous research of underreporting of the disease by factor of 2.7 with underestimations more likely for people identifying as Black and Latino. However, only 1.4% had a conclusive diagnosis of the disease by autopsy. Survival probabilities drop precipitously in the first few years after diagnosis with only 25% of the cohort surviving beyond 5 years.

